# Combining SENSE and reduced field-of-view for high-resolution diffusion weighted magnetic resonance imaging

**DOI:** 10.1186/s12938-018-0511-7

**Published:** 2018-06-15

**Authors:** Jisu Hu, Ming Li, Yakang Dai, Chen Geng, Baotong Tong, Zhiyong Zhou, Xue Liang, Wen Yang, Bing Zhang

**Affiliations:** 1 Suzhou Institute of Biomedical Engineering and Technology, Chinese Academy of Sciences, Suzhou, Jiangsu China; 2 Department of Radiology, Affiliated Drum Tower Hospital of Nanjing University Medical School, 321 Zhongshan Road, Nanjing, Jiangsu China

**Keywords:** Diffusion-weighted imaging, Reduced field-of-view, Single-shot echo-planar imaging, Parallel imaging, SENSE, g-Factor

## Abstract

**Background:**

In diffusion-weighted magnetic resonance imaging (DWI) using single-shot echo planar imaging (ss-EPI), both reduced field-of-view (FOV) excitation and sensitivity encoding (SENSE) alone can increase in-plane resolution to some degree. However, when the two techniques are combined to further increase resolution without pronounced geometric distortion, the resulted images are often corrupted by high level of noise and artifact due to the numerical restriction in SENSE. Hence, this study is aimed to provide a reconstruction method to deal with this problem.

**Methods:**

The proposed reconstruction method was developed and implemented to deal with the high level of noise and artifact in the combination of reduced FOV imaging and traditional SENSE, in which all the imaging data were considered jointly by incorporating the motion induced phase variations among excitations. The in vivo human spine diffusion images from ten subjects were acquired at 1.5 T and reconstructed using the proposed method, and compared with SENSE magnitude average results for a range of reduction factors in reduced FOV. These images were evaluated by two radiologists using visual scores (considering distortion, noise and artifact levels) from 1 to 10.

**Results:**

The proposed method was able to reconstruct images with greatly reduced noise and artifact compared to SENSE magnitude average. The mean g-factors were maintained close to 1 along with enhanced signal-to-noise ratio efficiency. The image quality scores of the proposed method were significantly higher (P < 0.01) than SENSE magnitude average for all the evaluated reduction factors.

**Conclusion:**

The proposed method can improve the combination of SENSE and reduced FOV for high-resolution ss-EPI DWI with reduced noise and artifact.

## Background

Diffusion-weighted magnetic resonance imaging (DWI) has shown great value both in clinical diagnosis and scientific studies. Single-shot echo-planar imaging (ss-EPI), which is widely used in current clinical DWI applications, has the advantages of short acquisition time, high signal-to-noise ratio (SNR) efficiency and less sensitivity to motion. Nevertheless, it also suffers from geometric distortion, image blurring and signal loss caused by the local field inhomogeneity of the static external magnetic field B0, for which imaging resolution is highly restricted in practice, even when parallel imaging is applied, such as sensitivity encoding (SENSE) [1] and generalized auto-calibrating partially parallel acquisitions (GRAPPA) [2].

To increase in-plane resolution without pronounced geometric distortion, reduced field-of-view (FOV) imaging has been developed by reducing the FOV along the phase-encoding (PE) direction and thus shortening the echo train length [3–8]. This technique is especially useful for high resolution diffusion imaging of specific anatomical structures like prostate [9–11], spinal cord [4, 12–16], optic nerve [17–19] and etc. For this goal further, an intuitive strategy is to combine parallel imaging with reduced FOV, which has been successfully applied in 7 T applications [20–22]. However, due to less field inhomogeneity in low field systems, the coil sensitivity maps are smoother than those at 7 T [23]. Moreover, the even smaller variance in the reduced FOV worsens the ill-conditioning of SENSE encoding matrix. Therefore, the combination of SENSE and reduced FOV often results in unacceptable noise and artifact level at low field strengths [24–26], which cannot be corrected by just averaging over the magnitudes of individual SENSE results.

In this paper, we propose a method for improving the combination of SENSE and reduced FOV imaging in ss-EPI DWI to produce images with reduced noise and artifact level. The phase variations among repeated acquisitions were incorporated into the coil sensitivities and all the imaging data can be jointly used to reconstruct the final image. In vivo experiment was carried out and the proposed method was applied for the acquired imaging data. Results show that the proposed method is effective in reducing noise and artifact level compared to the conventional SENSE magnitude average.

## Methods

### Theory

For Cartesian sampling trajectory, let *m* denote the number of folding pixels and *c* the number of coils, SENSE [1] can be formulated as **b** = **Sρ**, where **b** is the column vector of acquired aliased image pixels from all coil elements with length *c*, **S** the *c* × *m* sensitivity matrix, and **ρ** the vector of the *m* pixels in the to-be-reconstructed image. For simplicity, noise correlation is not considered here. The unfolded image can then be reconstructed through a pseudo-inverse by **ρ** = (**S**^**H**^**S**)^−**1**^**S**^**H**^**b** for each position in the aliased coil images. In matrix theory, the instability of matrix inversion is measured by the condition number. Correspondingly in SENSE, the g-factor is introduced and formulated as g_ρ_ = sqrt([(**S**^**H**^**S**)^−1^]_ρ,ρ_(**S**^**H**^**S**)_ρ,ρ_), which accounts for the SNR penalty imposed by the ill-conditioned matrix inversion. Hence, the g-factor is determined by the independency of coil sensitivity maps [26]. In reduced FOV, as the aliased pixel locations are closer than in the full FOV, the coil sensitivities in each row of **S** become similar, especially in low field systems. Consequently, matrix **S** will be very rank-deficient, which in turn results in high g-factors. In this case, noise in the sampled data can be much more amplified in the reconstructed images of reduced FOV than those of full FOV. This intrinsic problem makes the combination of reduced FOV and SENSE difficult in low field systems.

To solve this issue, it is proposed in this work to jointly reconstruct the final image from all the imaging data in the framework of SENSE. Due to the sensitivity to motion in DWI, images of repeated acquisitions usually have motion induced phase differences while the magnitudes generally remain unchanged. For this reason, conventionally these images are reconstructed individually and only the magnitudes are averaged for the final image (SENSE magnitude average, the default vender implementation). In the proposed method, though, the phase information is incorporated to construct the new formulation for all NSAs (number of signals averaged) like SENSE,

1 $$ \overline{{\mathbf{b}}} \, = \,\left[ {\begin{array}{*{20}c} {b_{1,1} } \\ \vdots \\ {b_{c,1} } \\ \vdots \\ \vdots \\ {b_{1,a} } \\ \vdots \\ {b_{c,a} } \\ \end{array} } \right] = \left[ {\begin{array}{*{20}c} {S_{1} ({\mathbf{r}}_{1} )\exp (i\varphi_{1} ({\mathbf{r}}_{1} ))} & \cdots & {S_{1} ({\mathbf{r}}_{m} )\exp (i\varphi_{1} ({\mathbf{r}}_{m} ))} \\ \vdots & \ddots & \vdots \\ {S_{c} ({\mathbf{r}}_{1} )\exp (i\varphi_{1} ({\mathbf{r}}_{1} ))} & \cdots & {S_{c} ({\mathbf{r}}_{m} )\exp (i\varphi_{1} ({\mathbf{r}}_{m} ))} \\ \vdots & \ddots & \vdots \\ \vdots & \ddots & \vdots \\ {S_{1} ({\mathbf{r}}_{1} )\exp (i\varphi_{a} ({\mathbf{r}}_{1} ))} & \cdots & {S_{1} ({\mathbf{r}}_{m} )\exp (i\varphi_{a} ({\mathbf{r}}_{m} ))} \\ \vdots & \ddots & \vdots \\ {S_{c} ({\mathbf{r}}_{1} )\exp (i\varphi_{a} ({\mathbf{r}}_{1} ))} & \cdots & {S_{c} ({\mathbf{r}}_{m} )\exp (i\varphi_{a} ({\mathbf{r}}_{m} ))} \\ \end{array} } \right]{\varvec{\uprho}} = {\overline{\mathbf{S}}}{\varvec{\uprho}} $$

where $\overline{{\mathbf{b}}}$ is the column vector of aliased image pixels from all coils and all NSAs with length ca, *S* the original coil sensitivity profiles, *φ* the phase of each NSA, ${\bar{\mathbf{S}}}$ the new ca × *m* sensitivity matrix with *a* counting the NSA, and **r** the pixel location. In this regard, data from all NSAs can be treated as sampled from the virtual coil elements with the new set of modulated coil sensitivity profiles defined by

2 $$ \bar{S}_{i} ({\mathbf{r}}) = S_{(i - 1)\bmod c + 1} ({\mathbf{r}})\exp (i\varphi_{{\text{floor} ((i - 1)/c) + 1}} ({\mathbf{r}})),\quad i = 1,2, \ldots ,\bar{c} $$

where *i* counts through the virtual coil elements. Therefore, the number of virtual coil elements $\bar{c}$ in Eq. (2) is *a* times of *c*. With the same unknowns but more equations, the linear equation system in Eq. (1) becomes more stable than the original SENSE for each individual acquisition and the g-factors can be kept in a reasonable level.

The phase information of each NSA can be obtained either from the image itself [27–29] or the navigator echo [30, 31]. In this work, total variation (TV) denoising [32] was applied for phase estimation formulated as

3 $$ \arg \mathop {\hbox{min} }\limits_{{x \in {\mathbf{C}}^{n} }} \left\{ {0.5||y - x||_{2}^{2} + \lambda ||\nabla x||_{1} } \right\} $$

where the first term measures the sum of squares error of the noisy signal y and the denoised x, and the second term represents the L1 norm of the gradient of x with the regularization term *λ*. Traditional SENSE was performed for each NSA followed by TV denoising according to Eq. (3), and the phase component was extracted from the denoised image. Then the virtual coil sensitivity profiles were constructed according to Eq. (2) for the proposed reconstruction.

### In vivo experiment

As a specific example of reduced FOV imaging, the previously proposed Improved Zoom FOV imaging (iZoom) [3] was used for data acquisition in this work. This method reduces the FOV along the PE direction with two dimensional selective excitation techniques and provides a new algorithm to improve the image uniformity. Hence, we name the proposed reconstruction as ZOOM-SENSE.

The in vivo experiment was designed to validate the effectiveness of the proposed method in reducing noise and artifact level compared to SENSE magnitude average at 1.5 T (Philips Healthcare, Multiva, Suzhou, China) using a 16-channel head-spine coil. Sagittal images of human cervical spinal cord from ten subjects were acquired after obtaining written informed consent and all human studies were performed under Institutional Review Board (IRB) approval from the local institution. The following parameters were kept the same in all the diffusion acquisitions: in-plane resolution = 1.4 × 1.4 mm^2^, slice thickness = 5 mm, 10 slices, PE direction = anterior/posterior (AP), TR = 3500 ms, NSA = 6, b = 0 s/mm^2^ and 3 diffusion encoding directions with b = 500 s/mm^2^, and scan time = 2.5 min. Specifically, the traditional full FOV (230 × 230 mm^2^) ss-EPI DWI with reduction factor (R) of 2 was first performed for comparison purpose. Then iZoom ss-EPI DWI was scanned using FOV of 230 × 80 mm^2^ with R = 1/1.5/2/2.5. Since the echo train lengths varied among these scans, the effective TE in full FOV DWI was 140 ms. For reduced FOV, the effective TEs for R = 1/1.5/2/2.5 were 124/99/86/79 ms. Finally, the corresponding T2-weighted images were obtained as the reference for evaluating the distortion levels of individual diffusion images.

All the diffusion imaging data were reconstructed using SENSE magnitude average first. The sensitivity maps were obtained by dividing the individual phased-array coil images by the corresponding body coil image in a reference scan. Then for reduced FOV data sampling with R = 1.5/2/2.5, ZOOM-SENSE was performed and the regularization term in TV denoising was set to be 0.1 empirically. The g-factor maps of both SENSE and ZOOM-SENSE were calculated along with the reconstructions to visualize the noise amplification. All the reconstructions were implemented offline in MATLAB (MathWorks, Natick, MA).

The relative SNRs (rSNR) of SENSE magnitude average or ZOOM-SENSE to full Fourier encoding [33] in reduced FOV for R = 1.5/2/2.5 were evaluated for each pixel according to

4 $$ rSNR = \frac{{\exp \left( {\frac{\Delta TE}{T2}} \right)}}{g\sqrt R } $$

where the numerator accounts for the signal increase with the TE reduction by Δ*TE*(TE_full-sampling_ – TE_reduced-sampling_), whereas the denominator includes the square root of R due to reduced data sampling and the g-factor penalty. The T2 relaxation time of white matter at 1.5 T was assumed to be 80 ms here. Both the spatial mean and minimum of the relative SNRs across the image were calculated for each R and reconstruction.

The diffusion images were also visually scored in terms of image quality, geometric distortion and artifact levels in a 10-point scale (10 for the best result). For each subject, the following methods were compared as detailed in Table 2. Hence, a total of 80 images were displayed in a random order and evaluated by two radiologists both with 3-year experience in MRI diagnosis. They were blinded to the acquisition parameters and reconstruction methods. The corresponding T2-weighted images were given for evaluating the distortion levels. The qualitative visual score measurements of SENSE magnitude average and ZOOM-SENSE results with the same acquisition parameters were compared using a Wilcoxon signed-rank test with a significance threshold of P < 0.05 in SPSS (SPSS Inc., Chicago, IL).

## Results

**Fig. 1:**
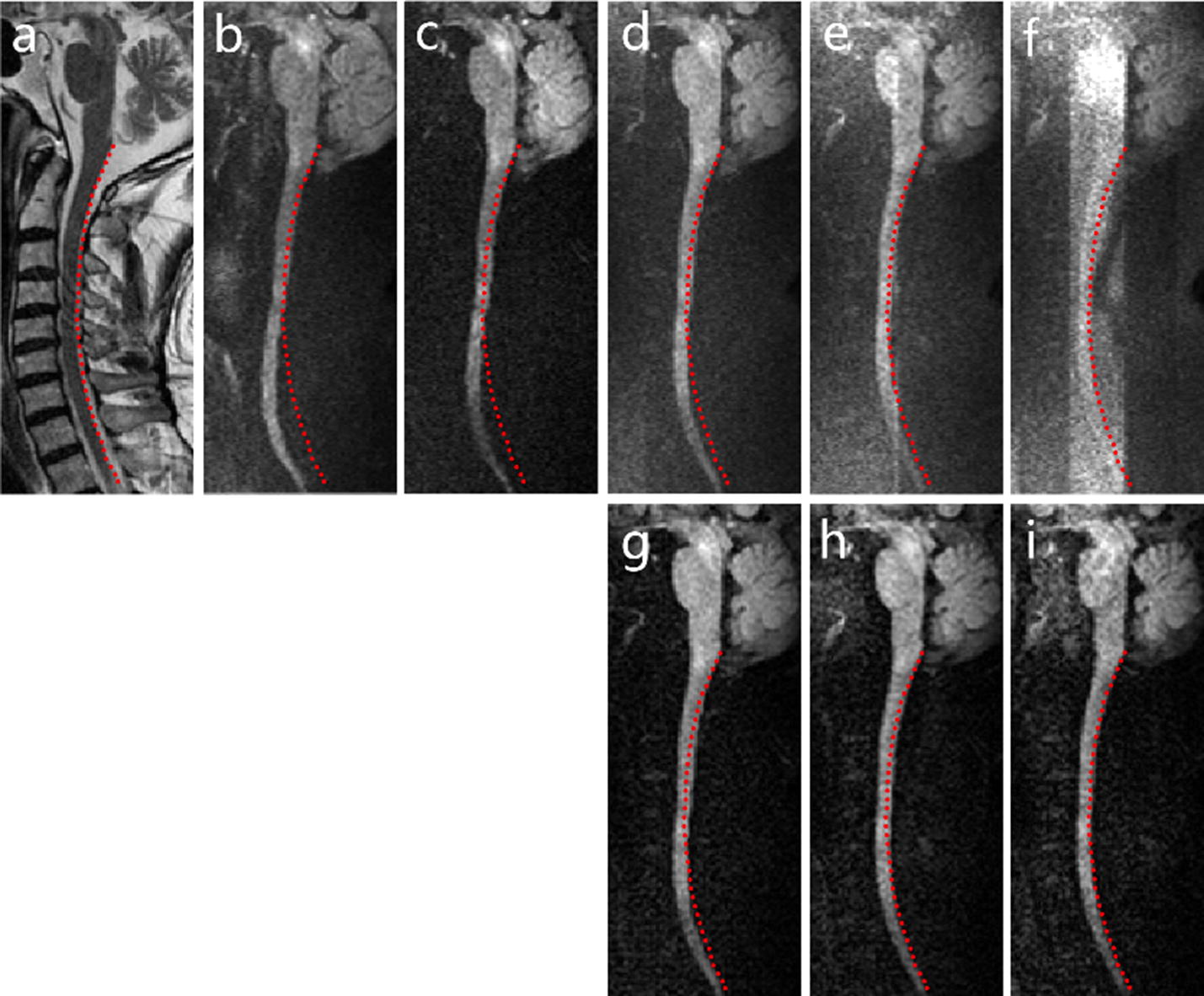
In vivo cervical spine images from one subject (male, aged 55). **a** The T2-weighted image shows slight cervical disc herniation. **b** The full FOV DWI with R = 2. **c** The fully sampled reduced FOV DWI. For R = 1.5/2/2.5, the SENSE magnitude average and ZOOM-SENSE results are shown in **d**–**f** and **g**–**i** respectively

Figure 1 shows all the diffusion images obtained using different acquisition parameters and reconstructions from one subject with slight cervical disc herniation (diffusion images show no damage in the spinal cord) along one diffusion direction. The red contour of the spine geometry is obtained from the corresponding T2-weighted image (Fig. 1a) and is overlaid onto all the presented diffusion images to evaluate the distortion levels. It can be seen that in the full FOV DWI with R = 2 (Fig. 1b) the spinal cord is most distorted from the true structure geometry among all the diffusion images. When using iZOOM excitation to reduce the FOV in the PE direction to 80 mm, the distortion level is reduced using fully sampled data (Fig. 1c) compared to the full FOV case and is further lowered gradually as R increases (Fig. 1d–f). Note that with R = 2 and 2.5, the geometry distortion is almost negligible and the spinal cord is well aligned with the red contour.

**Fig. 2:**
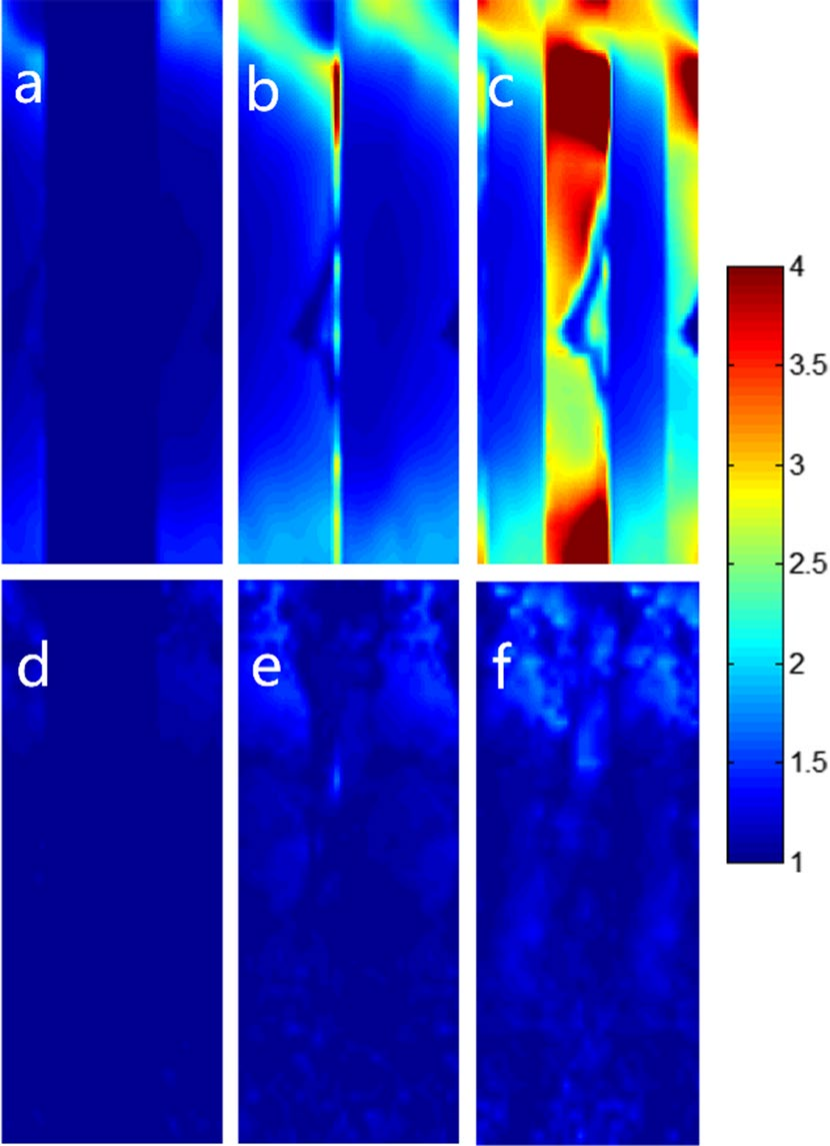
Comparison of g-factor maps of SENSE (**a**–**c**) and ZOOM-SENSE (**d**–**f**) for R = 1.5/2/2.5

Figure 1 also highlights the performance comparison of SENSE magnitude average and ZOOM-SENSE reconstructions in reduced FOV DWI. In SENSE magnitude average results, there is a distinct rise in the noise and artifact level as R increases (Fig. 1d–f). Especially at R = 2.5, the imaged object is covered by terribly heavy noise and artifact and the images are clinically unusable (Fig. 1f). In contrast, the anatomical details are shown more clearly in the corresponding ZOOM-SENSE results even using high reduction factors (Fig. 1g–i). The corresponding g-factor maps of SENSE and ZOOM-SENSE reconstructions are presented in Fig. 2, and the g-factors and relative SNRs are detailed in Table 1 for the three reduction factors. It can be observed that the maximum and mean g-factors of SENSE can reach up to 8.97 and 2.18 at R = 2.5, which will result in undesirable noise amplification, while those of ZOOM-SENSE can be kept no more than 2.22 and 1.11 in the same case. The relative SNRs demonstrate that the highly elevated g-factors outgrow the T2 effect in SENSE magnitude average reconstruction, whereas the SNR efficiency can be enhanced or at least maintained even at high reduction factors using ZOOM-SENSE.

**Table 1:** Comparison of SENSE magnitude average and ZOOM-SENSE in terms of g-factors and rSNRs

R	SENSE magnitude average				ZOOM-SENSE			
	g-Factor		rSNR		g-Factor		rSNR	
	Mean	Max	Mean	Min	Mean	Max	Mean	Min
1.5	1.08	1.97	1.0057	0.5662	1.01	1.4	1.0902	0.7948
2	1.43	4.35	0.8028	0.2612	1.04	1.68	1.0472	0.6762
2.5	2.18	8.97	0.5599	0.1238	1.11	2.22	0.9745	0.5001

**Fig. 3:**
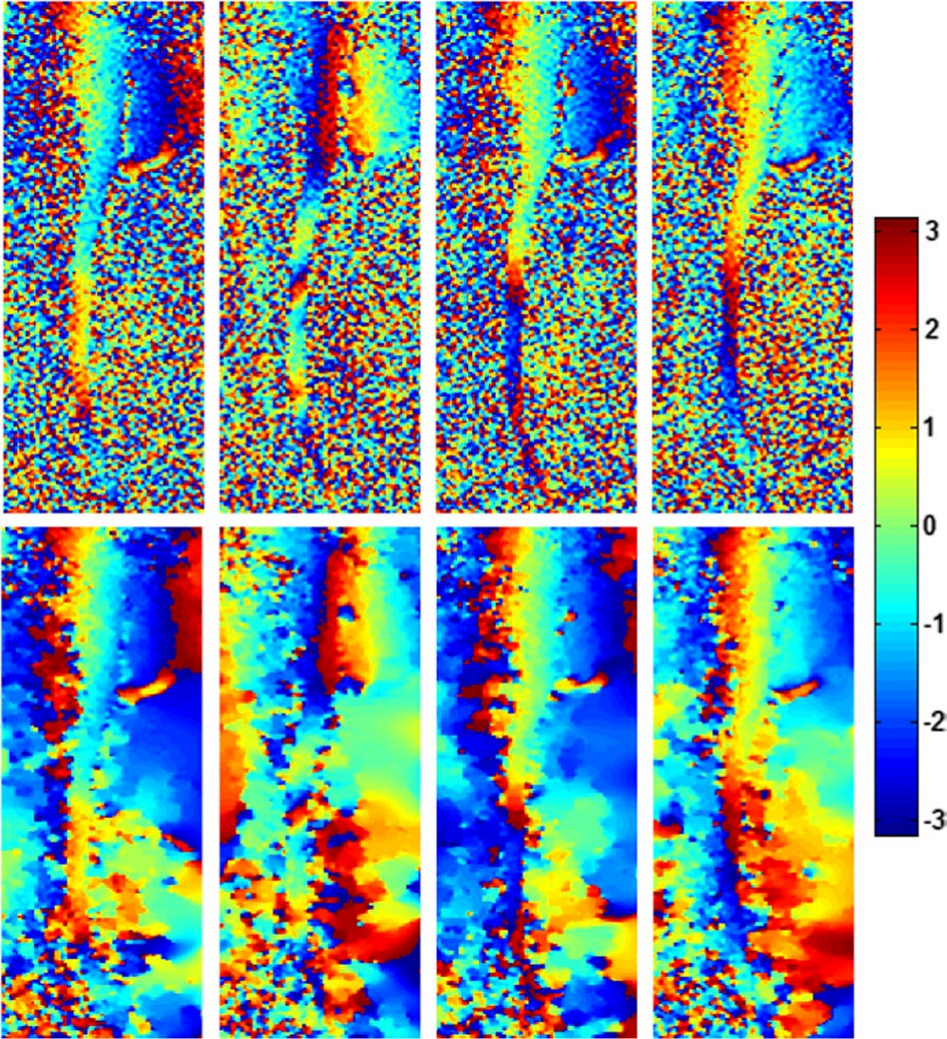
Phase maps from initial SENSE results before (upper row) and after (lower row) denoising for 4 representative NSAs for R = 2

Figure 3 shows the phase maps of initial SENSE results before and after denoising for 4 representative NSAs. Due to the problem of individual SENSE reconstruction in reduced FOV, the noise level is higher in the directly derived phase maps. After denoising, smoother phase maps can be obtained for the proposed reconstruction. More importantly, it is shown that the phase variations are obvious among different NSAs, which guarantees that imaging data from all NSAs can be combined effectively to perform ZOOM-SENSE reconstruction for reduced g-factors and better image quality.

Table 2 presents the imaging scores evaluated by the two reviewers for different combinations of scanning parameters and reconstruction methods. The first reviewer gives the highest mean score to the ZOOM-SENSE results at R = 1.5, while the second reviewer believes that the overall image quality is best in ZOOM-SENSE results at R = 2. For reduced FOV with R = 1.5/2/2.5, the image scores of ZOOM-SENSE are significantly higher than those of the corresponding SENSE magnitude average results (P = 0.007, 0.004 and 0.004 for the first reviewer, and P = 0.004, 0.005 and 0.004 for the second reviewer), which confirms the improvement of ZOOM-SENSE over SENSE magnitude average by radiologists.

**Table 2:** Image scores (mean ± STD) for the evaluated diffusion images by the two radiologists

FOV along PE direction (mm)	R	Reconstruction	Scores by reviewer 1 (mean ± STD)	Scores by reviewer 2 (mean ± STD)
230	2	SENSE magnitude average	5.80 ± 1.14	5.30 ± 0.48
80	1	SENSE magnitude average	6.50 ± 1.35	5.70 ± 0.48
80	1.5	SENSE magnitude average	7.00 ± 0.67	6.30 ± 0.48
80	1.5	ZOOM-SENSE	9.20 ± 0.79	8.30 ± 0.48
80	2	SENSE magnitude average	3.60 ± 0.84	4.10 ± 1.20
80	2	ZOOM-SENSE	8.70 ± 0.82	8.90 ± 0.57
80	2.5	SENSE magnitude average	1.60 ± 0.84	2.30 ± 0.48
80	2.5	ZOOM-SENSE	7.80 ± 0.42	7.80 ± 0.42

## Discussion and conclusion

In this study we proposed exploiting the phase inconsistency among NSAs to jointly perform SENSE-like reconstruction to improve the combination of reduced FOV excitation and parallel acquisition in ss-EPI DWI. To increase in-plane resolution without pronounced geometric distortion, the echo train length can be shortened intuitively by combining reduced FOV imaging and SENSE. However, compared to the full FOV case, closer locations of aliased pixels in reduced FOV can easily result in ill-conditioned inverse of SENSE encoding matrix and the corresponding g-factors will be much more elevated. In that case, even a small amount of noise in the sampled data can be amplified in the final image to an unacceptable extent. This happens when FOV is sufficiently small along with just slightly higher reduction factors. Moreover, due to the phase inconsistency among NSAs, individual SENSE results cannot be complex averaged. The in vivo experiment results confirm that the proposed ZOOM-SENSE reconstruction outperforms SENSE magnitude average in reducing noise and artifact level even when the SENSE results are severely corrupted. Note that the reduced TE by parallel acquisition will increase the SNR in the imaging data, but this advantage is hampered by the strong g-factor penalty introduced in traditional SENSE reconstruction. In ZOOM-SENSE, however, the overall g-factors are maintained close to 1, for which some anatomical details (e.g., the intervertebral discs) are better revealed with under-sampled data. More generally, although iZoom is used in this study for reduced FOV excitation, the proposed reconstruction can theoretically be combined with other small FOV imaging techniques. As well, it can potentially turn the combination of reduced FOV and SENSE into immediate practical use without additional hardware requirements or sequence modifications.

Despite the above advantages, it should be pointed out that the performance of ZOOM-SENSE is closely related mainly to two factors. The first is the extent of phase inconsistency among different NSAs. If more physiological motion is introduced during data acquisition, the phase maps will be very different from NSA to NSA, which brings stronger independency of the modulated coil sensitivity profiles in ZOOM-SENSE and results in more decreased overall g-factors. On the contrary, if ideally without NSA-to-NSA phase variations, the g-factor map in ZOOM-SENSE will remain the same as in traditional SENSE and performing ZOOM-SENSE will almost be identical to complex average of individual SENSE results. The second is the accuracy of phase estimation after initial SENSE results. Although in this study denoising is successfully used to get smooth phase maps for ZOOM-SENSE, the particularly high noise and artifact level is likely to hinder the phase estimation in some circumstances. When using higher reduction factors, the image object may be even buried in the noise and artifact in the initial SENSE results, making the phase estimation by denoising unreliable and leaving residual artifact on the images (e.g., the ZOOM-SENSE results for R = 2.5). In this case, an alternative way to obtain the phase information may be to acquire navigator echoes during the scan [30]. This might potentially outperform the phase estimation in this study but needs further investigations.

In conclusion, it is demonstrated that the proposed ZOOM-SENSE improves the combination of SENSE and reduced FOV at 1.5 T. By incorporating the phase information from all NSAs, all the imaging data can be treated as sampled from pseudo coil elements so that the matrix inversion becomes more stable than the original SENSE. Hence, the proposed method can produce images with decreased noise and artifact compared to SENSE magnitude average and it can be readily used in clinical applications.

## Abbreviations

DWI: diffusion-weighted magnetic resonance imaging
ss-EPI: single-shot echo planar imaging
FOV: field-of-view
SENSE: sensitivity encoding
GRAPPA: generalized auto-calibrating partially parallel acquisitions
SNR: signal-to-noise ratio
rSNR: relative signal-to-noise ratio
PE: phase-encoding
NSA: number of signal average
TV: total variation
iZOOM: improve zoomed FOV imaging
